# Supplementary material for: Crawling and Gliding: A Computational Model for Shape-Driven Cell Migration
Source: PLoS Comput Biol. 2015 Oct 21;11(10):e1004280. doi: 10.1371/journal.pcbi.1004280 (PMC4619082; doi:10.1371/journal.pcbi.1004280)
Supplement: S1 Code — (ZIP) [file pcbi.1004280.s012.zip › release/tst/doc/html/classGraphics-members.html]

Tissue Simulation Toolkit: Member List


Tissue Simulation Toolkit
 0.1.4.1


- Main Page
- Namespaces
- Classes
- Files

- Class List
- Class Hierarchy
- Class Members

Graphics Member List

This is the complete list of members for Graphics, including all inherited members.

|  |  |  |
| --- | --- | --- |
| BeginScene(void) | Graphics | inlinevirtual |
| EndScene(void) | Graphics | inlinevirtual |
| Field(const int \*\*f, int mag=1) | Graphics | inlinevirtual |
| GetXYCoo(int \*X, int \*Y)=0 | Graphics | pure virtual |
| Line(int x1, int y1, int x2, int y2, int colour)=0 | Graphics | pure virtual |
| Point(int color, int x, int y)=0 | Graphics | pure virtual |
| TimeStep(void) | Graphics | inlinevirtual |
| Write(char \*fname, int quality=-1)=0 | Graphics | pure virtual |
| XField(void) const | Graphics | inlinevirtual |
| YField(void) const | Graphics | inlinevirtual |
| ~Graphics(void) | Graphics | inlinevirtual |


---

Generated on Thu Aug 14 2014 22:04:01 for Tissue Simulation Toolkit by  

 1.8.6
